# Fatal systemic toxoplasmosis in a 3-month-old young tibetan goat (*Capra hircus*)

**DOI:** 10.1186/s12917-020-02641-8

**Published:** 2020-11-04

**Authors:** Silvia Pavone, Silvia Crotti, Deborah Cruciani, Nicoletta D’Avino, Jacopo Zema, Simone Morelli, Marco Gobbi, Laura Madeo

**Affiliations:** 1 Zooprophylactic Experimental Institute of Umbria and Marche “Togo Rosati”, Via G. Salvemini, 1, 06126 Perugia, Italy; 2 Faculty of Veterinary Medicine, University of Teramo, Teramo, Italy

**Keywords:** Genotyping, Goat, *Toxoplasma gondii*, Type II strain

## Abstract

**Background:**

Toxoplasmosis is one of the most common parasitic infections in both humans and animals. It is a frequent cause of abortion and stillbirth in intermediate hosts, especially sheep and goats but rarely causes fatal clinical form in adult animals.

**Case presentation:**

In contrast, the study reports an unusual fatal case of toxoplasmosis in a young goat naturally infected with type II strain of *Toxoplasma gondii*. A three-month-old female goat was presented with dyspnea and died few days later. Grossly, lungs were firm, edematous and mottled with disseminated whitish areas. Generalized lymphadenopathy was found. The histopathological examination showed necrotic interstitial bronchopneumonia and necrotizing lymphadenitis with intralesional free and clustered within macrophages tachyzoites of *T. gondii*. DNA extracted from lungs and lymph nodes was positive for *T. gondii* by a fast qPCR. PCR-RFLP analysis and sequencing of GRA6 gene showed that the isolated strains belonged to type II genotype.

**Conclusions:**

This is an unusual report of acute systemic toxoplasmosis caused by the type II strain of *T. gondii* with a fatal outcome in a young goat.

## Background

*Toxoplasma gondii* is an ubiquitous obligate intracellular protozoan parasite that occurs in most areas of the world. It is capable of infecting many species of warm-blooded animals and humans, and many different host cells [1, 2].

*Toxoplasma gondii* usually parasitizes the host without producing clinical signs and rarely causes severe clinical manifestations in nonpregnant animals. On the other hand, it is a frequent cause of abortion and stillbirth in intermediate hosts, especially sheep and goats [3, 4]. *Toxoplasma gondii* strains are categorized into three major clonal lineages (referred to as types I, II, III) according to virulence in outbreed mice [5]. Type I is rarer than the other strains and it is isolated mainly from human. It expresses virulence factors causing death in all the inoculated mice and therefore it is considered the virulent type [5]. Type II, the most commonly isolated, is non-lethal for inoculated mice; the animals develop a chronic infection with persistence of *T. gondii* as tissue cysts (avirulent type) [5]. Type III is rare and characterized by intermediate virulence [5]. Rare cases of systemic fatal toxoplasmosis in adult goats have been reported [3] and recently an outbreak in dairy goats characterized by abortion, stillbirth and death of adult goats occurred in Brazil [4].

The present article describes an unusual fatal systemic toxoplasmosis in a naturally infected tibetan young goat (*Capra hircus*) coming from a rural farm in central Italy.

## Case presentation

A dead three-month-old female goat was sent to the Diagnostics and Animal Welfare of Istituto Zooprofilattico Sperimentale dell’Umbria e delle Marche “Togo Rosati” for a necropsy examination. The breeder reported lethargy and dyspnea in the last days before the death. The animal came from a rural Tibetan goat farming in central Italy consisting of five adult goats (three female and two male) and three young goats including the dead one. A month before the young goat’s death, all farm goats have had access to the pasture of a neighbor frequented by cats.

A complete necropsy was performed. Representative samples were collected from organs with pathologic changes visible at the gross examination and fixed in 10% neutral buffered formalin for routine histological examination. Samples were embedded in paraffin wax, sectioned at 4 µm and stained with hematoxylin and eosin and with periodic acid–Schiff (PAS).

Samples from lung and thoracic and mesenteric lymph nodes were collected for molecular tests. The mother’s milk for the same test was also collected at a later date (15 days after the kid’s death). For lung and lymph nodes, 500 mg of tissue was homogenized in 1 ml of sterile saline solution with 5 mm stainless-steel beads using TissueLyser II (Qiagen, Hilden, Germany) at 30 Hz for 5 min. DNA was extracted using the QIAmp DNA mini kit (Qiagen) according to the manufacturer’s instructions. From mother’s milk, the DNA was extracted using the QIAGEN fluid protocol. All the samples extracted were examined by a multi-screening Fast qPCR assay for simultaneous detection of *T. gondii* and *Neospora caninum* [6]. A PCR-RFLP method on the GRA6 gene, a polymorphic single-copy gene which could easily differentiate the three distinct genotypes, was used for *T. gondii* strain typing [5, 7]. The digestion of the 791 bp PCR amplified product was performed using Tru 1I endonuclease (Fermentas, Germany) and the restriction fragments separated by electrophoresis on a 2,5% agarose gel and visualized under UV light. To confirm the data obtained, undigested PCR products were purified with QIAquick PCR purification Kit (Qiagen) and directly sequenced by Big Dye terminator v3.1 Kit (Thermo Fisher).

Spleen sample from young goat was also collected for molecular analysis for Border disease virus (BDV). The molecular genetic investigation was based on the coding 5′-UTR. Viral RNA was extracted with a QIAamp viral RNA mini kit (QIAGEN, Inc.). A 225-bp fragment of the 5′-UTR was amplified from randomly transcribed cDNA with the PBD1/PBD2 primers [8].

Seven blood samples were collected from live goats of the farm to evaluate the antibodies against *T. gondii*, *N. caninum* and Border disease using commercially available indirect ELISA kits (ID Screen *Neospora caninum* competition, IDvet, Grabels, France; ID Screen Toxoplasmosis Indirect Multi-species, ID.Vet, Grabels, France; IDEXX BVDV/MD/BDV p80 Protein Antibody Test Kit, IDEXX, Westbrook, Maine, USA) and the manufacturer’s recommendations.

Intestinal, pulmonary, lymph node and hepatic specimens were aseptically collected for bacteriological examinations. The samples were spread plated on MacConkey, mannitol salt agar and blood agar plates for aerobic incubation at 37 °C for 3 days. Then, biochemical reactions were performed for identification of the suspicious colonies. To anaerobic incubation, the samples were spread also on blood agar plate placed in the anaerobic jar with AnaeroGen reagent and incubated at 37 °C for 18–24 hours. Incubation was prolonged for 2 more days if no colonies were appeared at 24 hours.

A fecal sample (5 g) of dead goat was analyzed by the FLOTAC method [9] and examined with a light microscope at 100 × and 400 × magnification.

**Fig. 1:**
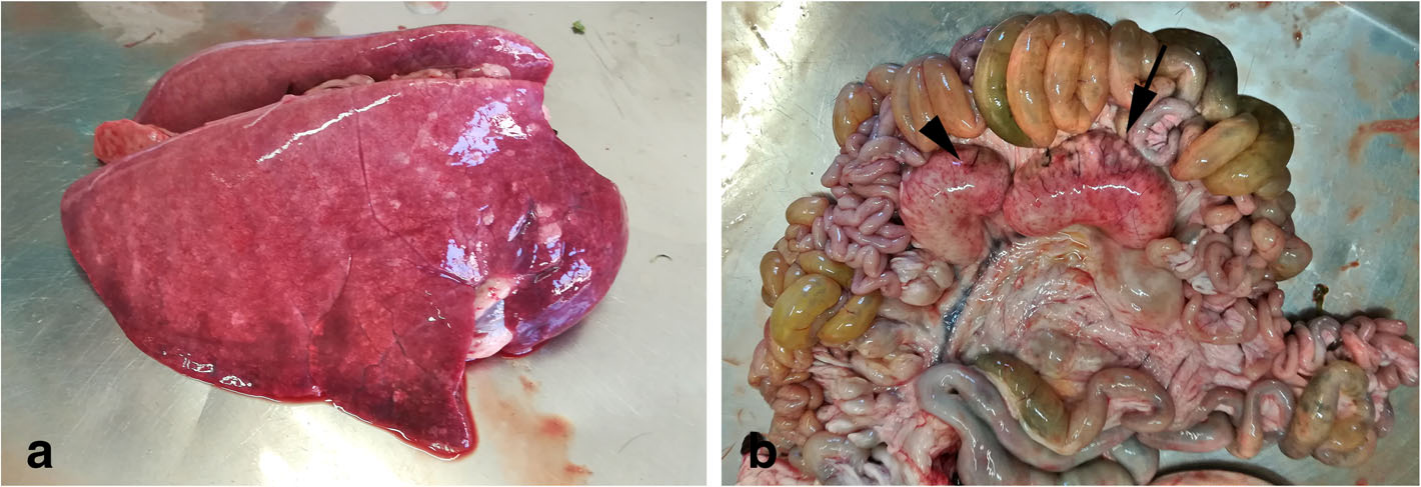
Macroscopic appearance of lungs and mesenteric lymph nodes. **a** Lungs appeared edematous and mottled with minute whitish disseminated areas. **b** Mesenteric lymph nodes were enlarged, mottled and characterized by grey or whitish areas (arrow) and hemorrhage (arrowhead)

Grossly, lungs were heavy, firm, edematous and mottled with disseminated whitish areas (Fig. 1a); abundant sero-sanguineous fluid was present at cut surfaces. Generalized lymphadenopathy was also found. In particular, enlarged mesenteric lymph nodes showed grey or yellowish areas and hemorrhage (Fig. 1b). Moderate catarrhal enteritis was also observed. No other macroscopic lesions were detected.

**Fig. 2:**
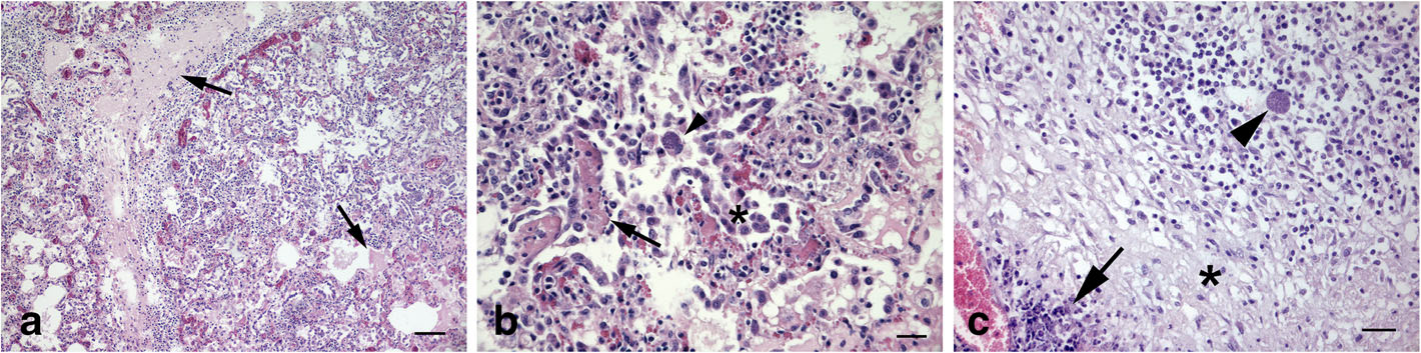
Microscopic appearance of lungs and mesenteric lymph nodes tissue. **a** Severe fibrin exudation in the interstitium and in alveolar lumina (arrows). Diffuse mixed inflammatory infiltrate of the interalveolar septa by lymphocytes, histiocytes, and scattered neutrophils. HE. Bar, 100 µm. **b** Necrosis of interalveolar septa with accumulation of eosinophilic and karyorrhectic debris (arrow) and type II pneumocyte hyperplasia (asterisk). Alveolar macrophages with intracytoplasmatic numerous basophilic structures morphologically consistent with *T. gondii* tachyzoites (arrowhead). HE. Bar, 50 µm. **c** Necrosis of lymphoid tissue (asterisk) and focus of the neutrophilic inflammatory infiltrate (arrow). Scattered macrophages with intracytoplasmatic *T. gondii* tachyzoites (arrowhead). HE. Bar, 25 µm

Histological examination of lungs showed alveolar septa and bronchial and bronchiolar interstitium thickening by fibrin, several macrophages and fewer neutrophils and lymphocytes. Necrosis of alveolar septa with accumulation of eosinophilic and karyorrhectic debris was also observed (Fig. 2a). Type II pneumocyte hyperplasia was diffusely found giving the appearance of a fetal lung (“fetalization” of lung) (Fig. 2b). Occasionally, interstitial and alveolar macrophages showed cytoplasmic cyst-like structures up to 40 µm in diameter containing numerous 2–3 µm, round, basophilic structures morphologically consistent with *T. gondii* tachyzoites (Fig. 2b). Similarly, both lymph nodes showed hemorrhage and necrosis with accumulation of karyorrhectic debris, fibrin and infiltration of macrophages and scattered neutrophils (Fig. 2c). Near and within the cytoplasm of large mononuclear cells *T. gondii* tachyzoites were detected. PAS stain showed negative results. Overall, the lesions suggested a severe, interstitial and necrotizing, diffuse, pneumonia and necrotizing lymphadenitis with intracellular parasitic elements consistent with *T. gondii*.

The tissue samples from lung and thoracic and mesenteric lymph nodes of young goat were positive for *T. gondii* by qPCR and the PCR-RFLP on GRA6 gene showed that the isolated strains belonged to type II genotype (identity 100%, E value 0.0). The consensus sequence obtained by the undigested PCR product was deposited in GenBank under the Accession Number MT321285. On the other hand, the qPCR for *T. gondii* from milk was negative as well as the *N. caninum* and the RT-PCR assay for BDV.

Anti-*Toxoplasma* antibody (IgG) ELISA test showed positivity results in the mother and sister goats of dead young goat. The other animals investigated were negative. Negative results were obtained also from anti-*Neospora* antibody (IgG) ELISA and anti-BDV (IgG) ELISA tests in goats of farm.

The bacteriological testing showed negative results. Copromicroscopic examination revealed 1728 coccidian oocyst per gram of faeces (OPG) and 864 gastrointestinal strongyle eggs per gram of faeces (EPG).

## Discussion and conclusions

Overall these results indicate a case of fatal systemic toxoplasmosis in a tibetan young goat (*Capra hircus*) infected with type II strain of *T. gondii*. It is well known that *T. gondii* causes abortion and stillbirth in intermediate hosts whereas rarely causes severe clinical manifestations in adult nonpregnant animals [3]. However, occasional cases of disease in adult goats were reported in literature. Three cases of systemic toxoplasmosis involving liver, kidneys and brain in adult goats were described by Dubey and Beattie (1988) [3] and, more recently, an outbreak in dairy goats characterized by lymphadenopathy, diarrhea, piloerection, weight loss, abortion, stillbirth and death of adult goats were reported in Brazil [4].

In the present case, severe interstitial, necrotizing, diffuse pneumonia and necrotizing lymphadenitis were detected. On the other hand, no relevant macroscopic lesions were detected in the intestine. However, in systemic cases of toxoplasmosis, infected hosts usually showed necrosis of intestines and mesenteric lymph nodes before other organs [1]. Therefore, it is likely that pathological intestinal conditions were grossly underestimated and lack of histopathological investigations on this organ did not allow a reliable detection of likely enteric lesions.

The molecular investigations showed that the present case of systemic toxoplasmosis is related to the type II strain of *T. gondii.* However, clonal type was assessed using only one genetic marker (GRA6) and in some cases this evaluation might produce a misleading genotype designation. Generally, genotype II is responsible for asymptomatic forms in mice in which the dissemination of tachyzoites is limited. Although, it is uncertain to what extent the genotype of the parasite directly affects the clinical severity of toxoplasmosis in other species, potential causes for immunosuppression were investigated in the young goat to clarify the role of parasite in the reported case. However, no disease known as cause of certain immunodeficiency disorders was detected. Only a moderate infections and infestations with coccidia and strongyles, respectively, were observed without apparent clinical signs and intestinal remarkable macroscopic lesions.

Given the age of the dead goat, the origin of the infection is not certain. Both infected mother’s milk or grazing pasture contaminated by infected cat faeces could have been potential sources. Molecular investigations on milk were negative. However, a previous study showed that the excretion of *T. gondii* in goat’s milk is irregular and shown up to 27 days after infection [10]. For these reasons, the present negativity of molecular analysis on milk may be not real and could be due to an untimely sampling of the milk (two weeks after the goat’s death) or to the intermittent presence of *T. gondii*. However, pasture contaminated by cat faeces containing *T. gondii* oocyst appears the more likely source of infection, as goat farm of the present study were allowed to graze in an areas frequented by cats potentially shedding oocysts.

In conclusion, this report describes an unusual case of acute systemic toxoplasmosis with a fatal outcome in a Tibetan young goat naturally infected with the type II strain of *T. gondii*.

Since toxoplasmosis represents a public health problem and causes economic losses to goat farms, increased knowledge about pathogenesis and the role of different *T. gondii* genotypes in the progress of the infection in this species would be desirable. Therefore, the systematic use of more genetic markers, or other methods, and larger genotyping studies may be useful to clarify if the severity of clinical forms are correlate to the genotype in goats.

## Data Availability

The datasets used and analyzed during the current study are available from the corresponding author on reasonable request.
